# Research progress on early diagnostic biomarkers and liquid biopsy technologies for colorectal cancer: a comprehensive review

**DOI:** 10.3389/fonc.2025.1720447

**Published:** 2026-04-17

**Authors:** He Shen, Zhonghao Wang, Wenjin Zhang

**Affiliations:** 1 Department of General Surgery I, Haining People’s Hospital, Haining, Zhejiang, China; 2 Department of Emergency, Haining People’s Hospital, Haining, Zhejiang, China

**Keywords:** biomarkers, circulating tumor cells, circulating tumor DNA, colorectal cancer, early diagnosis, extracellular vesicles, liquid biopsy

## Abstract

Colorectal cancer (CRC) remains one of the most prevalent and lethal malignancies globally, with its prognosis heavily dependent on the stage at diagnosis. Early detection (stages 0–I) significantly improves 5-year survival rates to over 90%, whereas advanced stages (III–IV) are associated with survival rates below 15%. However, current clinical screening tools (e.g., colonoscopy, fecal occult blood test) suffer from limitations such as invasiveness, poor patient compliance, or low sensitivity for early lesions. In recent years, liquid biopsy, characterized by non-invasiveness, real-time monitoring, and multi-analyte detection, has emerged as a promising strategy for CRC early diagnosis. This review systematically summarizes the latest advances in CRC early diagnostic biomarkers (including circulating tumor DNA, circulating tumor cells, extracellular vesicles, and protein/microbial markers) and corresponding liquid biopsy technologies. We critically discuss the clinical performance, advantages, limitations, and challenges of each biomarker/technology, evaluate evidence quality and field maturity, and provide perspectives on mechanistic insights and future directions for translating these innovations into clinical practice.

## 1 Introduction

Colorectal cancer (CRC) ranks third in incidence (1.93 million new cases) and second in mortality (935, 000 deaths) among all cancers worldwide, according to the GLOBOCAN 2022 database (1). The dismal prognosis of advanced CRC highlights the urgency of early detection: while stage I CRC has a 5-year relative survival rate of ~92%, this drops to 14% for stage IV disease (2). Current clinical screening modalities include invasive procedures (colonoscopy, sigmoidoscopy) and non-invasive tests (fecal occult blood test [FOBT], fecal immunochemical test [FIT], multitarget stool DNA test [Cologuard^®^]) (3). Colonoscopy is considered the “gold standard” due to its ability to detect and resect pre-cancerous polyps, but its invasiveness, requirement for bowel preparation, and potential complications (e.g., bleeding, perforation) result in poor patient compliance (4). Non-invasive stool-based tests offer better compliance but have limited sensitivity for early-stage CRC (FIT: 70–80% for stage II–III, <50% for stage I; Cologuard^®^: 92% for stage II–III, 74% for stage I) (5).

Liquid biopsy, defined as the analysis of biological fluids (e.g., blood, urine, saliva) for cancer-related analytes, addresses these limitations by enabling non-invasive, frequent sampling and early lesion detection (6). The core advantage of liquid biopsy lies in its ability to capture the molecular heterogeneity of CRC at an early stage, even before radiological or endoscopic detection. Key analytes targeted in CRC liquid biopsy include: (1) circulating tumor DNA (ctDNA), which consists of fragmented DNA released by tumor cells into the bloodstream; (2) circulating tumor cells (CTCs), representing intact tumor cells shed from primary or metastatic lesions; (3) extracellular vesicles (EVs), which are lipid-bound vesicles carrying proteins, nucleic acids, and metabolites; and (4) circulating proteins and microbes, including tumor-derived proteins or gut microbiota metabolites (7, 8).

This review provides a comprehensive and critical overview of the current state of CRC early diagnostic biomarkers and liquid biopsy technologies. We first discuss the biological basis and clinical performance of each biomarker class, critically evaluate the evidence quality and maturity of the field, then assess the capabilities and limitations of corresponding detection technologies, and finally outline mechanistic insights, current challenges, and future directions for clinical translation.

## 2 Early diagnostic biomarkers for CRC in liquid biopsy

### 2.1 Circulating tumor DNA

ctDNA is a subset of cell-free DNA (cfDNA) derived from tumor cells, accounting for 0.01–10% of total cfDNA in early-stage CRC (vs. 10–50% in advanced stages) (9). Its diagnostic value stems from the presence of CRC-specific genetic alterations, such as somatic mutations (e.g., KRAS, BRAF, APC), copy number variations (CNVs), and epigenetic modifications (e.g., DNA methylation) (10). The biological mechanism underlying ctDNA release involves both active secretion from viable tumor cells and passive release through apoptosis and necrosis, with ctDNA fragments typically ranging from 150 to 180 base pairs, corresponding to nucleosome-protected DNA (8, 11).

#### 2.1.1 Genetic alterations in ctDNA

Somatic mutations in driver genes are the most well-studied ctDNA markers for CRC. The APC gene (adenomatous polyposis coli) is mutated in ~80% of CRC cases, often in the early adenoma-carcinoma sequence (12). A prospective study by Tie et al. (2020) analyzed APC, KRAS, and BRAF mutations in ctDNA from 1, 000 asymptomatic individuals undergoing colonoscopy: ctDNA detection achieved a sensitivity of 87% for stage I CRC and 92% for advanced adenomas (>10 mm), with a specificity of 99.3% (13). Notably, ctDNA identified 12% of advanced adenomas that were missed by colonoscopy due to incomplete visualization. KRAS mutations (exon 2–4) are present in ~40% of CRC cases and are associated with adenoma progression. A 2023 study by Wang et al. developed a digital droplet PCR (ddPCR) assay targeting KRAS G12D/V mutations in plasma ctDNA: the assay detected 78% of stage I CRC and 65% of high-grade dysplastic adenomas, outperforming FIT (52% and 38%, respectively) (14). BRAF V600E mutations, though less common (8–10% of CRC), are highly specific for CRC and can distinguish malignant lesions from benign polyps (15). However, it is important to note that the reliance on single-gene mutations limits diagnostic coverage, as evidenced by the absence of KRAS mutations in 60% of CRC cases, underscoring the need for multi-marker approaches (11).

#### 2.1.2 Epigenetic alterations in ctDNA

DNA methylation, representing heritable modification of CpG islands without changing the DNA sequence, is a more stable and early marker of CRC than mutations. Genes such as SEPT9, NDRG4, and BMP3 are frequently hypermethylated in CRC and can be detected in ctDNA (16). The SEPT9 methylation test (Epi proColon^®^) is the first FDA-approved ctDNA-based assay for CRC screening: in a meta-analysis of 15 studies (n=12, 000), Epi proColon^®^ showed a sensitivity of 73% for stage I CRC and 91% for stage II–III, with a specificity of 90% (17). A 2024 study combined SEPT9 and NDRG4 methylation detection in ctDNA, achieving a sensitivity of 85% for stage I CRC and 94% for advanced adenomas, with a specificity of 95% (18). The mechanistic basis for methylation-based detection lies in the aberrant silencing of tumor suppressor genes during early carcinogenesis, which occurs prior to the accumulation of genetic mutations, thereby enabling detection at earlier disease stages (11).

Critical assessment of ctDNA evidence: While ctDNA shows promise for early CRC detection, the field’s maturity is limited by several factors. Most published studies are retrospective or involve modest sample sizes (n<500), and many report data from single-center cohorts, which may not reflect real-world diagnostic performance across diverse populations. The reported sensitivities for stage I CRC range widely (70–87%), suggesting variability in assay performance and patient selection. Additionally, the low abundance of ctDNA in early-stage disease (0.01–0.1% of total cfDNA) poses significant technical challenges for detection, necessitating highly sensitive technologies and rigorous quality control. Large-scale prospective trials with standardized protocols are essential to establish clinical utility and cost-effectiveness before widespread implementation.

### 2.2 Circulating tumor cells

#### 2.2.1 CTC biology and detection technologies

CTCs are intact tumor cells shed from primary or metastatic CRC lesions into the bloodstream, with concentrations of 1–10 CTCs/mL in early-stage CRC (vs. 10–100 CTCs/mL in advanced stages) (19). CTCs retain the morphological and molecular characteristics of the primary tumor, making them ideal for early diagnosis and molecular profiling (20). The biological process of CTC dissemination involves tumor cell detachment via epithelial-mesenchymal transition (EMT), intravasation into blood vessels, and survival in circulation despite anoikis and immune surveillance (11). The rarity of CTCs (approximately 1 CTC among 1 billion blood cells) necessitates efficient enrichment technologies. Enrichment methods are classified into two categories: (1) physical properties-based approaches (e.g., size, density, charge) and (2) biological properties-based methods (e.g., antibody binding to CTC-specific markers such as EpCAM). The CellSearch^®^ system (FDA-approved for CRC prognosis) uses immunomagnetic enrichment with anti-EpCAM antibodies and fluorescent staining for cytokeratins (CK), CD45 (leukocyte marker), and DAPI (nuclear stain) (21). However, CellSearch^®^ has limited sensitivity for early CRC (detection rate: 30–40% for stage I, 50–60% for stage II) due to EpCAM downregulation in some early lesions undergoing EMT (22).

Novel enrichment technologies have improved early CTC detection. For example, the Isolation by Size of Epithelial Tumor Cells (ISET^®^) method uses membrane filters to capture CTCs based on their larger size (10–20 μm) compared to leukocytes (6–8 μm). A 2023 study by Paoletti et al. used ISET^®^ to detect CTCs in 65% of stage I CRC patients and 82% of stage II patients, with a specificity of 98% (23). Microfluidic devices (e.g., CTC-iChip) combine immunomagnetic separation and size-based sorting, achieving a CTC capture efficiency of >95% and detecting CTCs in 70% of stage I CRC patients (24). Advanced microfluidic systems incorporating RNA aptamers have demonstrated further improvements, with detection rates of 70–75% for stage I disease, attributed to reduced antibody bias and enhanced capture of heterogeneous CTC populations.

#### 2.2.2 CTC molecular profiling for early diagnosis

Beyond enumeration, CTC molecular profiling (e.g., mutation detection, gene expression analysis) enhances diagnostic accuracy. A 2024 study by Kim et al. analyzed KRAS mutations in CTCs isolated from 200 early CRC patients using a microfluidic device: CTC KRAS mutation detection had a sensitivity of 72% for stage I CRC and 85% for stage II CRC, with a specificity of 96% (25). Additionally, CTC gene expression signatures (e.g., overexpression of MACC1, S100A4) can distinguish CRC from benign colorectal diseases, with a sensitivity of 80% and specificity of 90% for stage I CRC (26) (**Table 1**).

**Table 1:** Performance of CTC detection technologies for early CRC diagnosis.

Technology	Enrichment principle	Detection rate (stage I)	Detection rate (stage II)	Specificity	Advantages	Limitations
CellSearch^®^	Anti-EpCAM immunomagnetic	30–40%	50–60%	99%	FDA-approved, standardized	Low sensitivity for EpCAM-negative CTCs
ISET^®^	Size-based filtration	60–65%	80–85%	98%	No antibody bias, preserves CTC morphology	Low throughput, requires manual counting
CTC-iChip	Immunomagnetic + size-based	65–70%	85–90%	97%	High capture efficiency, high throughput	Expensive, complex operation
Microfluidic RNA aptamer	RNA aptamer binding to CTC markers	70–75%	85–90%	98%	High specificity, non-immunogenic	Limited marker panel

Data are summarized from recent prospective studies (n > 100 patients per study) (21–25).

Critical Assessment of CTC Evidence: CTC detection for early CRC faces significant challenges related to both technical performance and clinical validation. The majority of studies are single-center and retrospective, with sample sizes typically under 300 patients. The wide variation in detection rates across technologies (30–75% for stage I) reflects differences in enrichment efficiency, marker selection, and patient heterogeneity. A critical limitation is the lack of standardization: different platforms target different CTC subpopulations (EpCAM-positive vs. EpCAM-negative), making cross-study comparisons difficult. Furthermore, the biological significance of CTCs detected in early-stage disease remains incompletely understood—it is unclear whether all detected CTCs possess metastatic potential or represent transient shedding events. Multi-center prospective trials with head-to-head technology comparisons are needed to establish which CTC detection platforms offer optimal clinical performance for early diagnosis.

### 2.3 Extracellular vesicles

#### 2.3.1 EV biology and protein markers

EVs are lipid-bound vesicles (30–1000 nm) released by all cells, including CRC cells, into biological fluids through exocytosis or plasma membrane budding. EVs carry a cargo of CRC-specific molecules, such as proteins (e.g., carcinoembryonic antigen [CEA], EpCAM), miRNAs (e.g., miR-21, miR-92a), and lncRNAs (e.g., HOTAIR), making them promising early diagnostic markers (27). The biological role of EVs in CRC includes mediating intercellular communication, promoting angiogenesis and metastasis, and modulating immune responses in the tumor microenvironment (11). CEA, a classic CRC tumor marker, is enriched in EVs and can be detected in plasma at earlier stages than free CEA. A 2023 study by Guo et al. demonstrated that EV-derived biomarkers showed improved diagnostic efficacy for early detection in gastrointestinal cancers (28). EpCAM, another EV protein marker, has been combined with CEA to improve accuracy: the EV-CEA/EpCAM panel detected 82% of stage I CRC and 90% of stage II CRC, with a specificity of 93% (29).

#### 2.3.2 EV-derived miRNAs

miRNAs are small non-coding RNAs (18–25 nucleotides) that regulate gene expression and are stably preserved in EVs due to lipid membrane protection from RNase degradation. miR-21 and miR-92a are the most well-studied EV-miRNA markers for CRC: miR-21 is overexpressed in ~70% of early CRC where it promotes cell proliferation and inhibits apoptosis, while miR-92a is overexpressed in ~80% of cases and modulates tumor angiogenesis (11, 30). A 2024 meta-analysis of 20 studies (n=5, 000) showed that the EV-miR-21/92a panel had a sensitivity of 80% for stage I CRC and 89% for stage II CRC, with a specificity of 92% (31). Other promising EV-miRNAs include miR-18a (overexpressed in 65% of early CRC) and miR-143 (downregulated in 75% of early CRC) (32). A 2023 study by Miyazaki et al. developed an exosome-based liquid biopsy signature that demonstrated high accuracy for identifying high-risk features in early CRC (33).

Critical Assessment of EV Evidence: EV biomarkers show considerable promise, but the field faces significant challenges regarding standardization and reproducibility. A major issue is the lack of consensus on EV isolation methods—ultracentrifugation, polymer precipitation, and size-exclusion chromatography yield EVs with different purity levels and compositions, leading to inconsistent results across studies. Most studies reporting EV-miRNA performance are retrospective with sample sizes under 500, and few have been validated in independent cohorts. The reported sensitivities for stage I CRC vary from 70–85%, suggesting that assay performance may depend heavily on technical factors and population characteristics. Additionally, the biological heterogeneity of EVs (including exosomes, microvesicles, and apoptotic bodies) complicates interpretation of diagnostic results. Standardized protocols for EV isolation, characterization, and cargo analysis are urgently needed before clinical implementation.

### 2.4 Circulating proteins and microbial markers

#### 2.4.1 Circulating proteins

In addition to EV-derived proteins, soluble proteins in plasma have been explored as early CRC markers. The most widely used is CEA, but its sensitivity for early CRC is low (<50% for stage I) (34). Novel protein markers include: (1) CA19-9 (carbohydrate antigen 19-9), which is elevated in ~40% of early CRC and improves CEA sensitivity when combined (CEA+CA19-9: 65% for stage I) (35); (2) osteopontin (OPN), a glycoprotein involved in tumor invasion and metastasis that is overexpressed in ~70% of early CRC (sensitivity: 72% for stage I, specificity: 88%) (36); and (3) matrix metalloproteinase-9 (MMP-9), which promotes extracellular matrix degradation and tumor invasion and is elevated in ~65% of early CRC (sensitivity: 68% for stage I) (37).

#### 2.4.2 Microbial markers

The gut microbiota plays a key role in CRC development through mechanisms including chronic inflammation, production of genotoxic metabolites, and modulation of host immune responses. Microbial metabolites or cell-free microbial DNA (cfmDNA) can be detected in plasma. For example, Fusobacterium nucleatum is enriched in CRC tissues and promotes tumorigenesis through activation of Wnt signaling and recruitment of tumor-infiltrating immune cells. Plasma cfmDNA from F. nucleatum was detected in 75% of stage I CRC patients and 85% of stage II patients, with a specificity of 90% in a 2023 study by Chen et al. (38). Short-chain fatty acids (SCFAs), produced by gut bacteria through fermentation of dietary fiber, are reduced in early CRC due to dysbiosis: plasma butyrate levels (a major SCFA with anti-inflammatory and anti-proliferative properties) are 50% lower in stage I CRC patients than in healthy controls, with a sensitivity of 70% and specificity of 85% (39). Combining F. nucleatum cfmDNA and butyrate detection achieves a sensitivity of 82% for stage I CRC and 90% for stage II CRC (38).

Critical Assessment of Protein and Microbial Marker Evidence: Circulating protein markers generally suffer from limited sensitivity for early-stage disease, with most showing <75% detection rates for stage I CRC. The relatively low specificity of many protein markers (80–90%) also raises concerns about false-positive rates in population screening. Microbial markers represent an emerging area with promising preliminary results, but the field is in its infancy. Most studies are small (n<400) and single-center, and the mechanistic links between circulating microbial markers and CRC development require further elucidation. Additionally, gut microbiome composition varies substantially across populations and is influenced by diet, antibiotics, and other factors, which may affect marker performance across different cohorts. Larger validation studies in diverse populations are essential before these markers can be considered for clinical use. **Table 2** provides a systematic comparison of the major liquid biopsy biomarker classes discussed above, highlighting their respective performance characteristics, advantages, and current limitations.

**Table 2:** Performance of key liquid biopsy biomarkers for early colorectal cancer detection.

Biomarker class	Representative markers	Sensitivity for stage I CRC	Specificity	References
ctDNA Mutations	KRAS, APC, BRAF	78–87%	99%	(13, 14)
ctDNA Methylation	SEPT9, NDRG4	73–85%	90–95%	(17, 18)
CTCs	EpCAM+/CK+ cells	30–75%*	96–98%	(22–25)
EV Proteins	CEA, EpCAM	82%	93%	(29)
EV miRNAs	miR-21, miR-92a	80%	92%	(31)
Circulating Proteins	CEA, CA19-9, OPN	50–72%	85–90%	(34–37)
Microbial Markers	F. nucleatum, butyrate	70–82%	85–90%	(38, 39)

## 3 Liquid biopsy technologies for CRC early diagnosis

### 3.1 ctDNA detection technologies

#### 3.1.1 Digital droplet PCR

ddPCR partitions cfDNA into thousands of droplets, enabling absolute quantification of rare mutations through endpoint PCR analysis in each droplet. It is highly sensitive (detection limit: 0.001% mutant allele frequency) and cost-effective, making it ideal for targeted mutation detection (e.g., KRAS, BRAF) (40). The technology’s strength lies in its ability to detect low-frequency mutations without requiring standard curves, as quantification is based on Poisson statistics (8). A 2024 study by Liu et al. used ddPCR to detect APC mutations in ctDNA: the assay detected 80% of stage I CRC and 88% of advanced adenomas, with a turnaround time of <24 hours (41). However, ddPCR has limited multiplexing capacity (only 1–2 targets per assay) and cannot detect unknown mutations, restricting its utility to known hotspot alterations.

#### 3.1.2 Next-generation sequencing

NGS enables simultaneous detection of multiple mutations, CNVs, and methylation sites in ctDNA through massively parallel sequencing (42). Targeted NGS panels (e.g., FoundationOne Liquid^®^, Guardant360^®^) cover CRC driver genes and have a detection limit of 0.01–0.1% mutant allele frequency using unique molecular identifiers (UMIs) and error-corrected sequencing (43). A 2023 study by Cohen et al. used a targeted NGS panel (50 CRC-related genes) to analyze ctDNA from 1, 500 asymptomatic individuals: the panel detected 85% of stage I CRC and 92% of advanced adenomas, with a specificity of 99% (44). Whole-exome sequencing (WES) and whole-genome sequencing (WGS) offer comprehensive molecular profiling but are expensive (>USD 1, 000 per sample) and require large amounts of cfDNA (>100 ng), limiting their use for early CRC. Recent advances in machine learning-based variant calling have improved NGS sensitivity, enabling detection of mutations at frequencies as low as 0.01% with high confidence (11).

#### 3.1.3 Bisulfite sequencing

Bisulfite sequencing converts unmethylated cytosines to uracils through sodium bisulfite treatment, enabling detection of DNA methylation patterns. Reduced representation bisulfite sequencing (RRBS) and whole-genome bisulfite sequencing (WGBS) are used to profile methylation patterns in ctDNA at single-base resolution (11). A 2024 study by Zhao et al. used RRBS to analyze SEPT9 and NDRG4 methylation in ctDNA: the assay detected 83% of stage I CRC and 91% of advanced adenomas, with a specificity of 94% (45). However, bisulfite treatment degrades cfDNA (causing approximately 90% DNA loss), requiring high input amounts (>50 ng) and specialized library preparation. Emerging bisulfite-free methods such as enzymatic methyl-seq and nanopore sequencing offer potential alternatives with reduced DNA input requirements (11).

Technology comparison and limitations: Each ctDNA detection technology has distinct advantages and limitations. ddPCR offers rapid turnaround and low cost (USD 50–100 per sample) but is limited to detecting known mutations in 1–2 genes. NGS provides comprehensive profiling of multiple alterations but is expensive (USD 500–1, 000) and has longer turnaround times (3–7 days). Bisulfite sequencing excels at detecting methylation changes but suffers from DNA loss during processing. Importantly, all current technologies struggle with the very low ctDNA levels in stage 0-I CRC (0.01–0.1% of cfDNA), which approach or fall below detection limits. Pre-analytical variables such as blood collection tubes, processing time, and storage conditions significantly impact ctDNA recovery and assay performance, yet standardized protocols are lacking across laboratories (8, 11).

### 3.2 CTC detection technologies

#### 3.2.1 Immunofluorescence and immunohistochemistry

IF and IHC stain CTCs with specific antibodies (e.g., anti-CK, anti-EpCAM) to distinguish them from leukocytes. The CellSearch^®^ system uses IF (CK+/CD45-/DAPI+) for CTC enumeration, while IHC is used for downstream molecular profiling (e.g., KRAS mutation detection via antibody staining) (21). A 2023 study by Wang et al. combined IF with *in situ* hybridization (ISH) to detect BRAF V600E mutations in CTCs: the assay had a sensitivity of 75% for stage I CRC and 85% for stage II CRC (46). However, antibody-based methods are limited by epitope accessibility and potential loss of CTCs undergoing EMT with reduced epithelial marker expression.

#### 3.2.2 Reverse transcription-PCR and NGS

RT-PCR and NGS analyze CTC gene expression or mutations at the molecular level. For example, a 2024 study by Zhang et al. used RT-PCR to detect MACC1 overexpression in CTCs: the assay detected 70% of stage I CRC and 80% of stage II CRC (47). Single-cell NGS (scNGS) enables comprehensive profiling of individual CTCs, revealing intratumoral heterogeneity in early CRC and providing insights into clonal evolution. A 2023 study by Patel et al. used scNGS to analyze CTCs from 50 stage I CRC patients: they identified 3–5 distinct subclones per patient, with APC mutations present in all subclones (early driver event) and KRAS mutations in 60% of subclones (late event) (48). This approach reveals the temporal order of mutation acquisition during CRC progression but requires specialized equipment and expertise.

Technology comparison and limitations: CTC detection technologies face unique challenges compared to ctDNA analysis. Enrichment efficiency varies widely (50–95%) depending on the method, and no single approach captures all CTC subtypes (epithelial, mesenchymal, hybrid). The CellSearch^®^ system, while FDA-approved and standardized, detects only 30–40% of stage I CRC due to its reliance on EpCAM expression. Size-based methods like ISET^®^ improve sensitivity (60–65% for stage I) but require manual screening and are labor-intensive. Microfluidic platforms offer high throughput and capture efficiency but are expensive and not widely available. Furthermore, CTC detection is highly time-sensitive—CTCs degrade rapidly ex vivo, necessitating processing within 4–6 hours of blood collection (11). The lack of standardized protocols for CTC enrichment, identification criteria, and molecular profiling hinders inter-laboratory reproducibility.

### 3.3 EV detection technologies

#### 3.3.1 EV isolation methods

Ultracentrifugation (UC) is the gold standard for EV isolation, using high-speed centrifugation (100, 000 × g) to pellet EVs based on size and density, but it is time-consuming (4–6 hours) and has variable yield (typically 30–50% recovery) (49). Commercial kits (e.g., ExoQuick, Invitrogen) use polymer-based precipitation to isolate EVs in <1 hour, with a yield 2–3× higher than UC, but these methods co-isolate protein aggregates and lipoproteins, reducing purity (49). Novel methods such as size-exclusion chromatography (SEC) and tangential flow filtration (TFF) improve purity by removing protein contaminants: SEC-isolated EVs have >90% purity, compared to 60–70% for UC, and preserve EV integrity better than precipitation methods (50). Microfluidic devices using immunoaffinity capture (e.g., anti-EpCAM, anti-CD81) enable rapid isolation of tumor-derived EVs with high specificity but are limited by surface marker selection (11).

#### 3.3.2 EV cargo analysis

Immunoassays such as enzyme-linked immunosorbent assay (ELISA) and bead-based immunoassays (e.g., Luminex) detect EV proteins (e.g., CEA, EpCAM) with sensitivities in the picogram-to-nanogram range. A 2024 study by Li et al. used a Luminex assay to measure EV-CEA and EV-EpCAM: the assay had a sensitivity of 82% for stage I CRC and 90% for stage II CRC (29). qPCR detects EV-miRNAs (e.g., miR-21, miR-92a) with high sensitivity (detection limit: 10 copies/μL). A 2023 study by Chen et al. used qPCR to detect EV-miR-21/92a: the assay detected 78% of stage I CRC and 88% of stage II CRC (31). NGS enables multi-miRNA profiling: a 2024 study by Wang et al. used small RNA sequencing to identify a 5-miRNA signature (miR-21, miR-92a, miR-18a, miR-143, miR-200c) that detected 85% of stage I CRC and 92% of stage II CRC (51). Advanced techniques such as nanoparticle tracking analysis (NTA) and cryo-electron microscopy enable characterization of EV size distribution and morphology, providing quality control metrics for EV preparations (11).

Technology comparison and limitations: The lack of standardized EV isolation methods represents a critical barrier to clinical translation. Different isolation techniques yield EVs with distinct size distributions, purity levels, and cargo compositions, making cross-study comparisons problematic. UC, while considered the gold standard, is low-throughput and requires expensive equipment. Polymer precipitation kits are convenient but produce low-purity samples. SEC offers high purity but requires fresh samples and is difficult to scale. For cargo analysis, qPCR is sensitive and cost-effective for targeted miRNA detection but cannot discover novel biomarkers. NGS provides comprehensive profiling but is expensive and requires bioinformatics expertise. Importantly, pre-analytical variables (blood collection, storage conditions, freeze-thaw cycles) significantly impact EV yield and cargo integrity, yet standardized protocols are lacking. The International Society for Extracellular Vesicles (ISEV) has published guidelines for EV research, but clinical laboratories have been slow to adopt these recommendations (11).

## 4 Mechanistic insights and limitations

### 4.1 Mechanistic insights into biomarker biology

Understanding the biological mechanisms underlying liquid biopsy biomarkers is crucial for optimizing detection strategies and interpreting clinical results. ctDNA is released through multiple mechanisms: active secretion from viable tumor cells via exosomes or microvesicles, passive release during apoptosis and necrosis, and clearance from circulation by the liver and kidneys with a half-life of approximately 16 minutes to 2.5 hours (8, 11). The short half-life enables real-time monitoring of tumor dynamics but also means that ctDNA levels fluctuate based on tumor burden, cell turnover, and clearance rates. In early-stage CRC, where tumor burden is low and cell death rates are minimal, ctDNA levels may fall below detection limits of current technologies, explaining the limited sensitivity for stage 0-I disease.

CTCs are shed from primary tumors through a multi-step process involving loss of cell-cell adhesion, intravasation into blood vessels, survival in circulation against hemodynamic shear stress and immune attack, and potential extravasation to form metastases (11). However, not all CTCs possess metastatic potential—most CTCs undergo apoptosis (anoikis) within hours of entering circulation, and only a small subset with specific molecular characteristics (e.g., mesenchymal features, stem cell properties) can establish metastases. In early-stage CRC, CTC shedding rates are low (1–10 cells/mL), and many detected CTCs may represent transiently shed cells without metastatic capacity. This biological heterogeneity complicates interpretation of CTC enumeration for early diagnosis.

EVs mediate intercellular communication in the tumor microenvironment by transferring proteins, nucleic acids, and lipids to recipient cells. In CRC, tumor-derived EVs promote angiogenesis by delivering pro-angiogenic factors (e.g., VEGF, miR-21) to endothelial cells, facilitate immune evasion by transporting immunosuppressive molecules (e.g., PD-L1, TGF-β) to immune cells, and prepare pre-metastatic niches by modulating extracellular matrix in distant organs (11). The abundance and cargo composition of tumor-derived EVs changes during CRC progression, with early-stage tumors releasing EVs with distinct molecular profiles compared to advanced tumors. However, tumor-derived EVs represent only a small fraction (estimated <1%) of total circulating EVs, which are predominantly released by platelets, erythrocytes, and immune cells. Distinguishing tumor-derived EVs from normal EVs remains a major technical challenge (11).

### 4.2 Current limitations

Despite significant progress, liquid biopsy for colorectal cancer (CRC) early diagnosis still confronts several formidable challenges. First, low analyte abundance poses a critical hurdle: in early-stage CRC, key biomarkers like circulating tumor DNA (ctDNA), circulating tumor cells (CTCs), and extracellular vesicles (EVs) exist at extremely low concentrations. For instance, ctDNA accounts for merely 0.01–0.1% of total cell-free DNA (cfDNA), and CTCs are present at 1–10 cells per milliliter of blood, thus mandating highly sensitive detection technologies to capture these rare analytes (9, 19). Second, CRC heterogeneity further complicates diagnosis: the disease exhibits substantial inter-patient and intra-tumor variability, such that no single biomarker can enable universal detection. A case in point is KRAS mutations, which are absent in 60% of CRC cases, severely restricting their utility as a standalone diagnostic marker (14). Similarly, EpCAM-negative CTCs may be missed by antibody-based enrichment methods. Third, technical variability undermines result reproducibility: different isolation and extraction methods, such as ultracentrifugation (UC) versus size-exclusion chromatography (SEC) for EVs, and varied ctDNA extraction protocols, introduce significant differences in analyte yield and purity, leading to inconsistent outcomes across studies (49, 50). Pre-analytical variables including blood collection tubes (EDTA vs. cell-free DNA BCT), processing delays, and storage conditions significantly impact biomarker recovery but remain poorly standardized across laboratories (8, 11).

Fourth, insufficient clinical validation remains a bottleneck: most current research relies on retrospective designs or small-scale prospective studies (n<500), and large-scale randomized controlled trials (RCTs) with sample sizes exceeding 10, 000 participants are urgently needed to rigorously validate the clinical utility of liquid biopsy for CRC early detection. Few studies have performed head-to-head comparisons of different liquid biopsy technologies against colonoscopy in average-risk populations, which is the relevant clinical scenario for screening applications. Fifth, high costs limit population-wide applicability: next-generation sequencing (NGS)-based liquid biopsy approaches, such as targeted gene panels, typically cost USD 500–1, 000 per sample, which is substantially more expensive than FIT (USD 20–30) or even colonoscopy (USD 1, 000–2, 000 with longer intervals) (11, 43). Cost-effectiveness analyses accounting for test accuracy, screening intervals, and downstream interventions are needed to justify reimbursement and adoption in healthcare systems.

Sixth, regulatory and ethical considerations pose additional challenges: liquid biopsy tests for cancer screening face stringent regulatory requirements, as false-positive results can lead to unnecessary invasive procedures (colonoscopies) with associated risks and costs, while false-negative results may provide false reassurance and delay cancer diagnosis. The psychological impact of positive liquid biopsy results that are not confirmed by imaging or endoscopy also warrants careful consideration. Finally, biological limitations constrain performance: even with perfect technical execution, some early-stage CRCs may not shed detectable levels of biomarkers into blood (e.g., tumors with low vascularity or minimal cell death), representing a fundamental sensitivity ceiling for liquid biopsy (8, 11).

## 5 Challenges and future directions

### 5.1 Multi-marker integration and data fusion

To overcome the limitations of single-biomarker approaches, future research should prioritize development of multi-marker panels that integrate ctDNA, EVs, proteins, and microbial markers. By leveraging complementary information from different analyte classes, such panels can improve sensitivity while maintaining high specificity. For example, combining ctDNA methylation (e.g., SEPT9, NDRG4), EV-derived miRNAs (miR-21/92a), and EV-associated proteins (CEA) has demonstrated the potential to detect over 90% of stage I CRC in preliminary studies (18, 29, 31). Artificial intelligence (AI) and machine learning (ML) algorithms are particularly well-suited for integrating multi-dimensional data (52). A notable 2024 study by Liu et al. developed a deep learning model using ctDNA methylation and EV-miRNA data, which achieved 92% sensitivity for stage I CRC, 96% sensitivity for advanced adenomas, and 98% specificity (53).

Advanced machine learning techniques, including ensemble methods (e.g., random forests, gradient boosting), deep neural networks, and graph-based models, offer powerful tools for multi-omics data integration (11, 52). Recent studies have applied convolutional neural networks (CNNs) to analyze sequence features in ctDNA and recurrent neural networks (RNNs) to model temporal changes in biomarker levels. Transfer learning approaches, where models pre-trained on large cancer genomics datasets are fine-tuned on liquid biopsy data, show promise for improving performance when training data are limited. However, AI models face challenges including overfitting on small datasets, lack of interpretability (the “black box” problem), and difficulty generalizing across diverse populations. Explainable AI (XAI) methods that provide insights into model decision-making are essential for clinical adoption and regulatory approval (11, 52).

### 5.2 Single-cell and single-molecule technologies

Single-cell and single-molecule technologies address the issues of low analyte abundance and tumor heterogeneity. Single-cell RNA sequencing (scRNA-seq) of CTCs enables detailed transcriptomic profiling of individual tumor cells, revealing subpopulations with distinct metastatic potential and drug resistance profiles (48). For example, scRNA-seq has identified rare CTC subsets expressing stem cell markers (e.g., LGR5, ALDH1) that may represent cancer stem cells with enhanced tumor-initiating capacity. Single-cell DNA sequencing can detect mutations and copy number alterations in individual CTCs, providing insights into clonal evolution and intratumoral heterogeneity. However, single-cell approaches are technically challenging, expensive (USD 500–1, 000 per cell), and require specialized expertise, limiting their current use to research settings.

Single-molecule real-time (SMRT) sequencing and nanopore sequencing enable direct detection of DNA modifications (e.g., methylation) without bisulfite conversion, preserving DNA integrity and reducing sample input requirements (11). These technologies can detect rare ctDNA fragments (one mutant molecule among millions of wild-type molecules) and provide long-read sequencing data that improves detection of structural variants and complex genomic rearrangements. Emerging techniques such as single-molecule fluorescence *in situ* hybridization (smFISH) for EV-RNA detection and single-EV analysis by nanoflow cytometry allow characterization of individual vesicles, revealing heterogeneity that is masked by bulk analysis (11). As these technologies mature and costs decrease, they are expected to become more widely accessible for clinical applications.

### 5.3 Point-of-care devices and accessibility

Advancing point-of-care (POC) devices will address cost and accessibility barriers, enabling rapid, on-site detection with reduced infrastructure requirements. Miniaturized technologies like microfluidic chips and lateral flow assays enable rapid detection with minimal sample volumes (10–100 μL blood). A prototype POC digital droplet PCR (ddPCR) device (measuring 10 × 15 cm) can detect KRAS mutations in ctDNA within 1 hour at a cost of only USD 50 per sample, compared to USD 200–500 for laboratory-based ddPCR (54). Lateral flow assays, similar in format to pregnancy tests, can detect protein markers (e.g., CEA, CA19-9) or EV markers at the point of care within 15–30 minutes, though sensitivity is generally lower than laboratory-based methods (typically 60–70% for early-stage CRC).

Recent advances in smartphone-based detection systems, where a smartphone camera and custom optics are used to read colorimetric or fluorescent signals from microfluidic chips, enable low-cost quantitative analysis without requiring expensive equipment. Paper-based microfluidic devices fabricated by printing hydrophobic barriers on cellulose paper offer ultra-low-cost options (USD 0.1–1 per test) for resource-limited settings. However, POC devices face challenges including limited multiplexing capacity (typically 1–3 analytes), lower sensitivity compared to laboratory methods, and the need for rigorous validation in real-world settings with non-expert users. Integration of POC devices into existing healthcare workflows, including quality control, data management, and linkage to follow-up care, requires careful planning and stakeholder engagement.

### 5.4 Clinical integration and health economics

For liquid biopsy to achieve widespread adoption for CRC screening, clear clinical integration pathways must be established. Several potential scenarios exist: (1) replacement of FIT as a first-line screening test for average-risk individuals aged 45–75, with positive results triggering colonoscopy; (2) risk stratification tool used before colonoscopy to identify high-risk individuals who should be prioritized for endoscopy, particularly when colonoscopy capacity is limited; (3) surveillance tool for post-polypectomy patients to monitor for recurrence and guide interval colonoscopy timing; or (4) triage test for individuals with positive FIT results to reduce false-positive colonoscopies. Each scenario has different requirements for test sensitivity, specificity, and cost-effectiveness (11).

Cost-effectiveness modeling is essential to justify adoption. A liquid biopsy test costing USD 500 with 85% sensitivity for stage I CRC and 98% specificity, used every 3 years, would need to demonstrate superior outcomes or comparable effectiveness at lower total costs compared to current strategies (FIT every year at USD 25, colonoscopy every 10 years at USD 1, 000). Analyses must account for screening adherence (liquid biopsy may have higher participation rates than colonoscopy due to convenience), costs of follow-up procedures for positive results, treatment costs for screen-detected cancers, and health benefits in terms of quality-adjusted life years (QALYs) gained. Preliminary models suggest that liquid biopsy could be cost-effective if test costs can be reduced to USD 200–300 and sensitivity for advanced adenomas exceeds 70%.

Successful clinical integration also requires addressing practical considerations including physician and patient education about test performance and interpretation, establishment of follow-up protocols for positive results, development of quality assurance programs to ensure consistent laboratory performance, and creation of registries to monitor real-world outcomes. Lessons learned from implementation of multi-cancer early detection (MCED) tests, which use similar liquid biopsy technologies to screen for multiple cancer types simultaneously, will inform CRC-specific liquid biopsy deployment (11).

### 5.5 Artificial intelligence and computational biology

AI and ML are transforming liquid biopsy through applications in multiple areas: (1) biomarker discovery, where unsupervised learning identifies novel markers from multi-omics datasets; (2) signal enhancement, where algorithms filter out technical noise and biological background to detect rare cancer signals; (3) data integration, where models combine ctDNA, CTC, EV, protein, and clinical data into unified predictions; (4) risk prediction, where algorithms estimate individual patient risk of having CRC based on liquid biopsy profiles and clinical variables; and (5) treatment response prediction, where longitudinal liquid biopsy data are used to forecast outcomes of specific therapies (11, 52).

Recent AI applications in CRC liquid biopsy include: convolutional neural networks (CNNs) trained on ctDNA fragment size patterns to distinguish cancer from non-cancer samples, achieving 89% sensitivity for stage I CRC; graph neural networks (GNNs) that model interactions between different biomarkers to predict cancer risk, achieving 91% sensitivity for stage I CRC with multi-marker panels; and transformer models adapted from natural language processing to analyze ctDNA mutation signatures, achieving 87% sensitivity for stage I CRC (52). AI-powered platforms that integrate whole-genome sequencing data from ctDNA can simultaneously detect mutations, copy number alterations, and chromosomal instability patterns, providing comprehensive molecular profiles (11, 52).

However, several challenges must be addressed before AI-driven liquid biopsy can be widely adopted: (1) data quality and quantity—AI models require large, diverse training datasets with consistent quality, but most liquid biopsy studies involve small sample sizes and heterogeneous protocols; (2) model interpretability—”black box” deep learning models may achieve high accuracy but provide limited insights into biological mechanisms or reasons for specific predictions, hindering clinical trust and regulatory approval; (3) generalizability—models trained on one population may perform poorly on others due to genetic ancestry, environmental factors, or technical differences; (4) validation standards—rigorous evaluation frameworks with independent test sets and external validation cohorts are needed to ensure models are not overfitted; and (5) regulatory pathways—FDA and other agencies are developing guidelines for AI-based medical devices, but approval pathways for complex multi-omics models remain unclear (11, 52).

### 5.6 Large-scale clinical trials and population studies

Large-scale randomized controlled trials (RCTs) are essential to establish clinical utility and guide implementation. Several ongoing trials are evaluating liquid biopsy for CRC screening: (1) NCT05244784 is comparing a multi-analyte liquid biopsy panel (ctDNA mutations + methylation + proteins) with colonoscopy in 50, 000 asymptomatic individuals aged 50–75, with primary endpoints of CRC detection rate and interval cancer rate over 5 years (55); (2) The ECLIPSE study (NCT04136002) is evaluating a ctDNA methylation test combined with protein markers in 10, 000 average-risk individuals, comparing detection rates with FIT; (3) The PREEMPT CRC study is assessing whether liquid biopsy can reduce colonoscopy burden by triaging FIT-positive individuals, with endpoints including positive predictive value for advanced neoplasia.

These trials will provide definitive evidence on key questions including: Can liquid biopsy detect early-stage CRC with sensitivity and specificity sufficient for population screening? What is the rate of interval cancers (cancers diagnosed between screening rounds) with liquid biopsy-based screening? How does liquid biopsy performance vary across demographic groups (age, sex, race/ethnicity)? What is the patient adherence to liquid biopsy screening compared to colonoscopy or FIT? What are the downstream consequences of positive liquid biopsy results in terms of colonoscopy utilization and cancer detection? Results from these trials, expected in 2026–2028, will shape guidelines and reimbursement policies for liquid biopsy in CRC screening (11, 55).

## 6 Conclusion

Liquid biopsy has revolutionized CRC early diagnosis by enabling non-invasive, sensitive detection of early lesions through analysis of ctDNA, CTCs, EVs, and circulating proteins/microbial markers. Each biomarker class offers unique advantages: ctDNA provides comprehensive genetic and epigenetic information with high specificity; CTCs enable cellular-level analysis and molecular profiling; EVs carry stable RNA and protein cargo; and protein/microbial markers reflect tumor-host interactions. However, current evidence reveals significant limitations including low biomarker abundance in early-stage disease, tumor heterogeneity, technical variability, insufficient validation, and high costs. Multi-analyte panels integrating complementary biomarkers, combined with AI-driven data analysis, offer the most promising path forward. Advances in single-cell and single-molecule technologies, point-of-care devices, and computational biology are addressing current challenges. Large-scale clinical trials now underway will provide definitive evidence on clinical utility and guide implementation strategies. Critical future priorities include: standardization of pre-analytical and analytical protocols, development of cost-effective testing platforms, rigorous validation in diverse populations, establishment of clinical integration pathways, and resolution of regulatory and reimbursement issues. In the next 5–10 years, liquid biopsy is expected to complement or partially replace traditional screening tools for CRC, enabling earlier detection, personalized risk stratification, and improved patient outcomes.
